# Drowning physiology and the effective factors on drowning in Guilan's beaches and swimming pools 

**Published:** 2019-07

**Authors:** Mojtaba Ahmadpour-Roudesari

**Affiliations:** ^*a*^Applied Research Office, Guilan Province Police Command, Rasht, Iran.

## Abstract

**Background:**

Drowning is considered as one of the serious public health problems, especially in Guilan and in summer season. The study of physiology of drowning allows us to become acquainted with specific physiological stages that disrupt the sensitive ionic balance of the cell. With proper understanding, we can design suitable therapeutic interventions. It is obvious that the sequence of events when drowning in freshwater is different from that of salt water, and the study of effective factors in this field can be of great help in providing preventive strategies.

**Methods:**

In order to investigate the effective factors on drowning, 150 samples of drowning and near-drowning reports were reviewed in coastal waters and pools of Guilan province from 2012 to 2017. This is a descriptive-analytic study of cross-sectional type. For all patients, a checklist including age, sex, drowning and swimming skill was prepared. Data were analyzed using SPSS software and chi-square tests.

**Results:**

Of all drowning cases in beaches of Guilan, 102 ones were men (68%) and 48 women (32%), and the most cases of drowning were in the age group of 20-24 years old with 39 cases (26%). Summer was the season with highest incidence in drowning (n=132, 88%). Also, 54 ones (45.3%) had the ability to swim deep in the sea.Drowning in swimming pools of Guilan was recorded in 84 men (56%) and 66 women (44%), and the most frequent age group was 28-32 years old with 59 cases (39.3%). Summer was the season with highest drowning cases (n=124, 82.6%). Also, 54 cases (36%) had the ability to swim in the deep pool area.

**Conclusions:**

Obviously, the sequence of events of drowning is different in freshwater from that of drowning in salt water because fresh water is hypotonic to blood, while sea water is hypertonic. Drowning in sea water has a different sequence compared with drowning in sea water (containing 3.5% salt) which is hypertonic to blood (0.9% salt). Various studies have concluded that spontaneous resuscitation of people with near drowning and drowning or asphyxia (without breath), is possible which requires a series of initial measures. Moreover, according to the results of this study, drowning incidence was higher in men and younger children and men with a low educational level. Insufficient skill, joking with friends and lack of familiarity with warning signs are very important factors in drowning. Considering that many tourists are interested in swimming in the Caspian Sea during the summer season and young boys and people are at the higher risk of drowning, informing them about the dangers and causes of drowning and the first aid training seem to be essential.

**Keywords:**

Drowning, Sea, Swimming Pools

